# Characterization of Different Cable Ferrite Materials to Reduce the Electromagnetic Noise in the 2–150 kHz Frequency Range

**DOI:** 10.3390/ma11020174

**Published:** 2018-01-23

**Authors:** Adrian Suarez, Jorge Victoria, Antonio Alcarria, Jose Torres, Pedro A. Martinez, Julio Martos, Jesus Soret, Raimundo Garcia-Olcina, Steffen Muetsch

**Affiliations:** 1 Department of Electronic Engineering, University of Valencia, Burjassot 46100, Spain; jose.torres@uv.es (J.T.); pedro.a.martinez@uv.es (P.A.M.); julio.martos@uv.es (J.M.); jesus.soret@uv.es (J.S.); raimundo.garcia@uv.es (R.G.-O.); 2 Würth Elektronik eiSos GmbH & Co. KG, Waldenburg 74638, Germany; jorge.victoria@we-online.de (J.V.); antonio.alcarria@we-online.de (A.A.); steffen.muetsch@we-online.de (S.M.)

**Keywords:** cable ferrite, electromagnetic interferences, low frequency emissions, nanocrystalline, relative permeability, insertion loss

## Abstract

The gap of standardization for conducted and field coupled electromagnetic interferences (EMI) in the 2–150 kHz frequency range can lead to Electromagnetic Compatibility (EMC) problems. This is caused by power systems such as Pulse Width Modulation (PWM) controlled rectifiers, photovoltaic inverters or charging battery units in electric vehicles. This is a very important frequency spectral due to interferences generated in a wide range of devices and, specifically, communication problems in the new technologies and devices incorporated to the traditional grid to convert it into a Smart Grid. Consequently, it is necessary to provide new solutions to attenuate this kind of interference, which involves finding new materials that are able to filter the electromagnetic noise. This contribution is focused on characterizing the performance of a novel material based on nanocrystalline and comparing it to most common material compositions such as MnZn and NiZn. This research is carried out from the point of view of the manufacturing process, magnetic properties and EMI suppression ability. This last item is carried out through two analysis procedures: a theoretical method by determining the attenuation ratio by measuring impedance parameter and proposing a new empirical technique based on measuring directly the insertion loss parameter. Therefore, the main aim of this characterization process is to determine the performance of nanocrystalline compared to traditional cable ferrite compositions to reduce the interferences in this controversial frequency range. From the results obtained, it is possible to deduce that nanocrystalline cable ferrite provides the best performance to filter the electromagnetic noise in the 2–150 kHz frequency range.

## 1. Introduction

Standardization regarding disturbances in the 2–150 kHz frequency range has been progressing in recent years and can be classified into the conducted electromagnetic interferences (EMI) range as power quality harmonics (2–9 kHz) and CISPR A band (9–150 kHz) [1,2]. Nevertheless, there is still a gap in the standardized limits that can result in interferences between devices at the 2–150 kHz band. Electromagnetic disturbances located from 2 kHz to 150 kHz are known as supraharmonics and they are generated by most mains related to power conversion applications, like battery electrical vehicles chargers [3] or photovoltaic systems [4]. Furthermore, these disturbances have also been detected in other kinds of systems related to household appliances such as washing machines [5] and lighting installations [6].

One of the reasons for this problem is the lack of knowledge and controllability of the low voltage harmonic grid impedances and the high sensitivity of devices to generate electromagnetic disturbances. These interferences can be caused by internal circuit topology or the presence of certain neighbor devices connected to the same grid [7]. An important problem caused by this kind of electromagnetic noise is related to the systems and devices included in the electrical network to convert it into a Smart Grid [8]. This is a significant area because one of the main consequences of a Smart Grid is a strong increase in use of electronics to include the intelligence in the power system. This means that the correct function of electronic equipment needs to be taken into account for implementing a smart power grid, which integrates intelligently, the actions of all users in order to promote renewable energy sources and efficiently use of electricity.

Specifically, most of the smart meters used to determine the power consumption are typically equipped with communication interfaces to transmit the readings to the network operator or Power Company through Power Line Communication (PLC) protocol (9 to 95 kHz). By lacking proper legislation, the frequency band 2–150 kHz has been used as the ‘garbage’ band during the last years [9], generating as a result, interferences in the PLC communication. Therefore, it has to be guaranteed that the unintentional emissions or interferences are lower than the intentional emission by the PLC in order to ensure its proper functionality in the 2–150 kHz frequency band.

Thereby, it is essential to ensure the electromagnetic compatibility (EMC) in the 2–150 kHz frequency band in terms of radiated and conducted disturbances. Consequently, new solutions in terms of EMI filtering have to be proposed with the aim of reducing the electromagnetic noise, which can cause disturbances in this spectral band. An interesting component that is widely used by designers to meet EMC compliance requirements is ferrite.

Ferrite is a generic term for a class of nonconductive ceramics that are based on materials such as oxides of iron, cobalt, nickel, zinc or magnesium. Ferrite manufacturers have even designed their own compositions, this implies that there is a large variety of ferrite combinations.

Ferrites provide an inexpensive way of coupling high-frequency resistance into a circuit without introducing power loss at dc or affecting any low-frequency signals present. Traditionally, ferrites have been most effective in providing attenuation of unwanted signals above 1 MHz because these components can provide the suppression of high-frequency oscillations, common-and differential-mode filtering, and the reduction of conducted and radiated emissions [10,11]. The material used to make a ferrite determines the frequency range of applicability. They are available in many different configurations, such as thru-hole beads on leads, surface-mount beads, sleeve cable cores, flat cable cores, snap on cores, toroids, etc. This contribution is focused on analyzing the performance of cable cores, also known as cable ferrites, because these components slip over a conductor lead and, hence provide a solution against EMI without the need of electronic circuit redesign.

One of the best advantages of ferrite cores is their ability to attract magnetic flux and, therefore, to suppress electromagnetic noise without grounding. In this way, when a cable is passed through a ferrite core, the magnetic field generated by the cable is concentrated inside the core and it is converted to heat and dissipated by the magnetic loss of the ferrite. Cable ferrite EMI suppressors are applied widely in electronics and telecommunications since they are able to suppress interference over a wide frequency range. They are generally focused on filtering interferences in the Megahertz band and the most common compositions are MnZn and NiZn variety [12,13]. The use of MnZn cable ferrites is usually limited to the higher Kilohertz and very low Megahertz region, whereas ferrite cores made of NiZn material work in a broadband frequency range which can cover several hundreds of Megahertz and even frequencies about 1 GHz. 

The target of this research is to determine the performance of a novel cable ferrite component based on nanocrystalline (NC) material with the aim of studying its suitability for solving or reducing EMC problems in the frequency range of 2–150 kHz. Some researchers have looked into the use of NC compositions [14] to make other EMC components, such as the common-mode-choke (CMC) in order to filter low frequency electromagnetic interferences. This is because NC cores allow the volume of the component can be reduced by 50–80% [15] and provide higher permeability and insertion losses (A) at low frequencies [16]. Thereby, a prototype of NC core is characterized and compared with MnZn and NiZn traditional ceramic cores in order to determine the effectiveness of each material in that controversial frequency range. This is firstly performed by measuring and analyzing typical core parameters provided by datasheet product such as relative permeability and impedance response. Subsequently, an experimental characterization procedure is described and carried out with the aim of determine the insertion loss parameter of each cable ferrite. This parameter is usually estimated by means of the cable ferrite impedance [11]; however, this contribution proposes an experimental method, which simulates supraharmonics generated to determine the attenuation ration that NC, MnZn and NiZn cable ferrites can provide at CISPR A band. Therefore, a comparison between the insertion loss parameter calculated theoretically from the cable ferrite impedance and the attenuation measured with the experimental setup is performed.

## 2. Materials and Methods

### 2.1. Cable Ferrites Characterization

Cable cores belong to ferromagnetic materials and it can be divided into three groups: ceramics, metals and composite materials. MnZn and NiZn cable ferrites characterized in this research are included into ceramic materials, whereas the NC core is classified as metal. The cable ferrites that have been characterized in this contribution have been selected because they have similar dimensions and, at the same time, they are composed with different materials. Thereby, it is possible to evaluate their performance to solve electromagnetic noise problems found below 150 kHz. These parameters are shown in Table 1.

Ceramics are also known as polycrystalline materials and, although they do not belong to metals, they can contain metal oxide such as manganese oxide or zinc oxide. The main characteristics of ceramics are their strong adhesion forces, heat resistant, hardness, brittleness and high resistance to pressure. The manufacturing process is performed by mixing the oxides, calcining the material obtained, breaking the calcined mixture, transform it into granular, pass these grains through a pressing process, synthetized the material and, finally, cover it with an epoxy resin to reduce its conductivity. One of the main advantages of ceramics manufacturing process is the possibility of creating cores with many different shapes.

**Figure 1:**
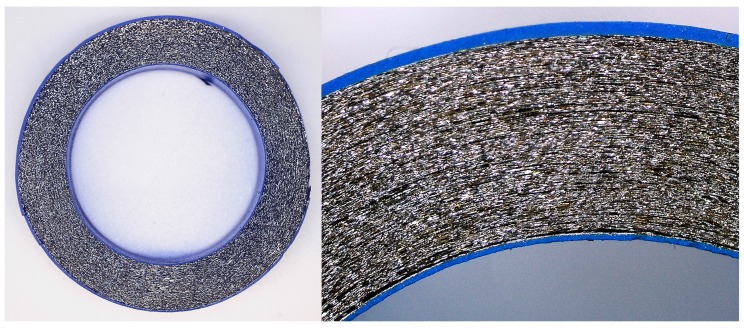
Cross section of nanocrystalline (NC) cable ferrite internal layers.

NC is a magnetic material which is being increasingly used in other types of applications where low frequency electromagnetic noise can become a problem, such as energy storage, current sensors or electronic filters [17]. The main advantage of NC material is because of the material’s intrinsic properties it can be used to design more reduced components with better magnetic properties for low frequency applications. NC cable ferrite, as opposed to MnZn and NiZn, is not a solid core since it is formed by a rolled up about 20 µm metal film. Figure 1 shows the cross section of the NC core in which it is possible to observe the metal film and the coated material used to press and protect it. The external coated layer is very important due to the metal film being very brittle, thus, any breaking could generate discontinuities in the material and deteriorate its performance. Consequently, one limitation of this material is its ability to manufacture different core shapes because the metal film cannot be cut and it is not possible to manufacture air gap or snap cores.

**Figure 2:**
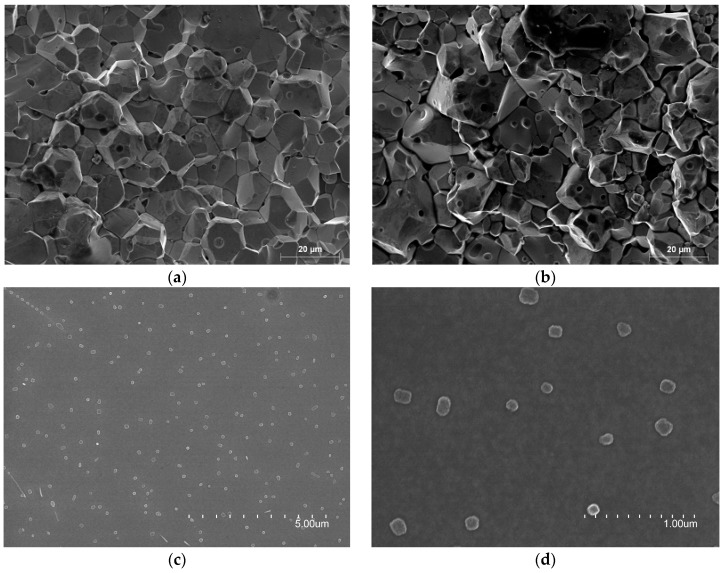
SEM photographs of ceramics and nanocrystalline core materials: (**a**) MnZn material composition; (**b**) NiZn material composition; (**c**) NC material composition; (**d**) NC material composition zoom in.

The NC production process is much more complex than the ceramic because, firstly, it consists of heating the material at 1300 °C in order to melt it. This material composition is mainly based on Fe, which has been added Nb, Mo and Cu amorphous metals, as well as metalloids Si and B in order to form the glass and stabilize the amorphous structure [18,19]. Once the material is converted into liquid melt, this is poured on a cooling wheel which is spinning around at 100 km/h with the aim of reducing the material temperature at a cooling rate of 10^6^ K·s^−1^ [16]. This high cooling rate is necessary to generate the amorphous structure and define the thickness of the film about 20 µm. Subsequently, the film is rolled up to form the cylindrical core and it is subjected to an annealing process in which this is heated between about 500 °C and 600 °C to achieve the nanocrystalline state. During this step, the material is submitted to magnetic fields in order to improve its magnetic properties. This crystallization process of Fe-(Si,B) glasses with the addition of small amounts of amorphous metals lead to the creation of ultrafine grain sizes of typically 10–15 nm [20]. Figure 2 shows three micrographs of the samples obtained using scanning electron microscopy (SEM, Hitachi, Tokyo, Japan). In these photographs, it is possible to observe the difference between the ceramic materials where the grain size is in the order of tens of micrometers and NC material where the grain size is in the order of nanometers.

One of the most important parameters that describes the material′s capacity to absorb electromagnetic noise is the permeability (µ), which is defined by micro-structural factors such as, the distribution of grain size, domain wall, spin rotation, the amount of pore and the homogeneity of chemical composition inside grain [21]. The permeability parameter relates the magnetic flux density to the magnetic field in a defined medium, thus, when a ferromagnetic material such a cable ferrite is placed in a magnetic field, the magnetic flux is concentrated in it. Thereby, the parameter that determines the factor, by which the induction (B) is modified when a material is introduced, is the relative permeability (µ_r_) [22]. The losses of the magnetic flux can be quantified by separating it into its complex form so that the real component is related to the reflection or inductive part and the imaginary component provides the losses or absorption part [23,24]. The real part is represented by µ′ which defines the magnetic flux that the material is able to reflect and the imaginary part corresponds to µ′′ which describes the core material effectiveness to absorb the magnetic noise.

The behavior of these parameters depends on the material internal composition and it is usually represented versus the frequency. One way of knowing the performance of the three different cable ferrites materials mentioned above, is through examining the permeability parameter. Ceramic materials are defined by a high saturation and a permeability between 100 and 15,000 depending on its internal characteristics [25]. In the case of the MnZn cable ferrite, it represents a very important soft magnetic material because of its high initial magnetic permeability, saturation magnetization, electrical resistivity and low power losses [26]. Likewise, its practical range of frequencies is the medium frequency range that is going from some tens of kHz to a few MHz [27]. With regard to NiZn ferrites, they are one of the most versatile soft magnetic materials, especially suitable for high-frequency applications due to their high magnetic permeability from tens of MHz to hundreds of MHz and its larger cut-off frequency compared to another composition like MnZn [21]. In terms of composition, both ceramic materials are mostly based on iron oxide (Fe_2_O_3_) with a proportion of about 70%. In this way, NiZn cable ferrite contains a higher amount of Zn to improve the cut-off frequency with the aim of attenuate electromagnetic noise at higher frequencies [28,29]. Furthermore, Cu is introduced in the NiZn composition in order to reduce the firing temperature to ease the manufacturing process as well as improve electromagnetic properties at high frequencies [30]. On the other hand, NC presents a much higher initial permeability than ceramic materials because it has been annealed and subjected to a magnetization process. This improvement of magnetic properties produces a very significant increase in the permeability, reaching values about 100,000 [16].

**Figure 3:**
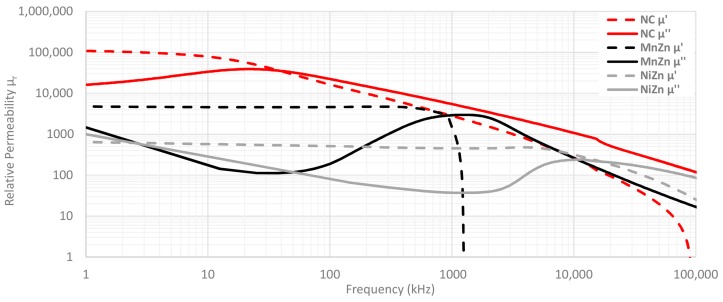
Complex Relative Permeability of NC, MnZn and NiZn cable ferrites compositions.

Figure 3 represents the relative permeability traces of NC, MnZn and NiZn cable ferrite compositions split into real and imaginary components. Frequency resonance is an important value in this graph as it is the point where real and imaginary parts crosses and, thus, the material changes its dominant behavior. The values represented in this graph have been measured with specific and calibrated equipment by following two setups depending on the frequency range selected. From 1 MHz to 100 MHz, the complex relative permeability has been obtained with a E4991A Material Analyzer equipment (Keysight, Santa Rosa, CA, USA) together with its 16454A Magnetic Material Text Fixture. The 16454A makes it possible to carry out accurate permeability measurements of round-shaped magnetic materials due to the construction of this fixture creating one turn around the core without magnetic flux leakage. Thereby, it is possible to obtain complex permeability direct readouts of complex permeability. This equipment does not allow us to measure permeability from 1 kHz to 1 MHz, so a second method is employed in which relative permeability of magnetic material is obtained from the self-inductance of a round-core that has a closed loop. This second setup provides the value of effective permeability derived from the inductance measurement results by using Equations (1) and (2). This procedure is carried out with a E5061B Vector Network Analyzer (Keysight, Santa Rosa, CA, USA) together with a Terminal Adapter 16201A (Keysight, Santa Rosa, CA, USA) and the Spring Clip Fixture 16092A (Keysight, Santa Rosa, CA, USA) by rolling three turns in the cable ferrite component in order to evaluate the inductance with respect to the ends of the wire [31].

The relative permeability parameter and impedance of a certain cable ferrite are conditioned by, on the one hand, the imaginary component is related to the loss or resistive component and, on the other hand, the real component corresponds to the inductance component. The mathematical expressions which demonstrate this are given by:

(1) $$µ\prime =\frac{\ell L}{{µ}_{0}N^{2}A}$$

(2) $$µ\prime \prime =\frac{\ell R}{{µ}_{0}N^{2}\omega A}$$

where ℓ corresponds to average magnetic path length of toroidal core [m], µ_0_ is the permeability of the air, N is the number of turns given with a cable to carry out the measure, A is the cross-sectional area of the toroidal core [m^2^], ω is the angular frequency, R is the resistive or loss component and L is the inductance component of the cable ferrite.

**Figure 4:**
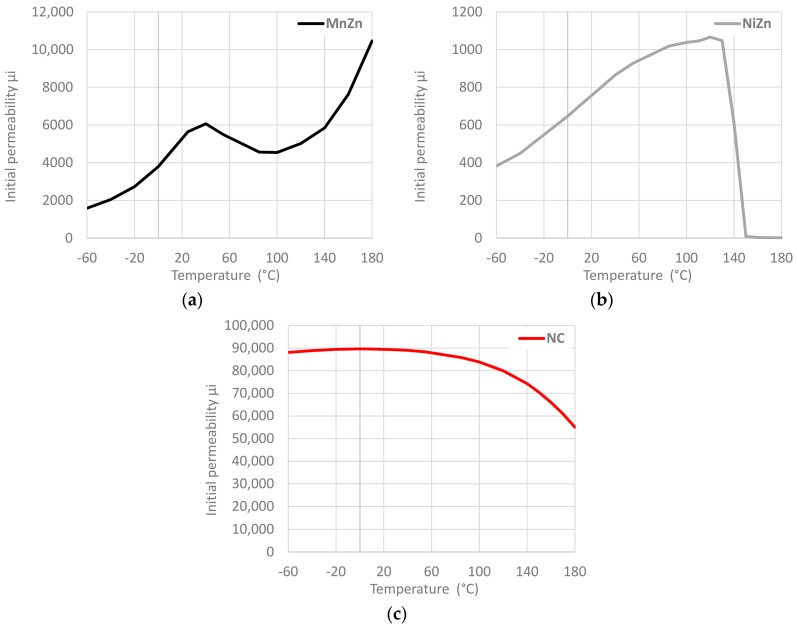
Initial permeability versus temperature for three different cable ferrites: (**a**) MnZn; (**b**) NiZn; (**c**) NC.

One of the most used parameters for comparing different EMI core materials is the initial permeability (µ_i_). This parameter describes the relative permeability of a material at low values of B (below 0.1 T). Low flux has the advantage that every cable ferrite can be measured without risk of saturation and, therefore, it is easier to compare different compositions. According to IEC 60401-3, µ_i_ is defined using closed magnetic circuits for f ≤ 10 kHz, B < 0.25 mT, T = 25 °C. Another important parameter to find out the performance of a certain magnetic material is the Curie temperature as it indicates at which temperature it loses its ferromagnetism [16]. Figure 4 shows the change of initial permeability over a temperature range from −60 °C to 180 °C. This characterization has been performed with the equipment E4980A Precision LCR Meter (Keysight, Santa Rosa, CA, USA) that provides the initial permeability through measuring the real part of complex permeability parameter [11] and carrying out temperature sweep. Thereby, in order to evaluate the component, it has been introduced into the CST-Temperature Test Chamber series T-40. From these graphs, we can observe the great stability of the initial permeability of NC cable ferrite compared to MnZn and NiZn. NC initial permeability remains at values higher than 80%, up to about 150 °C.

**Figure 5:**
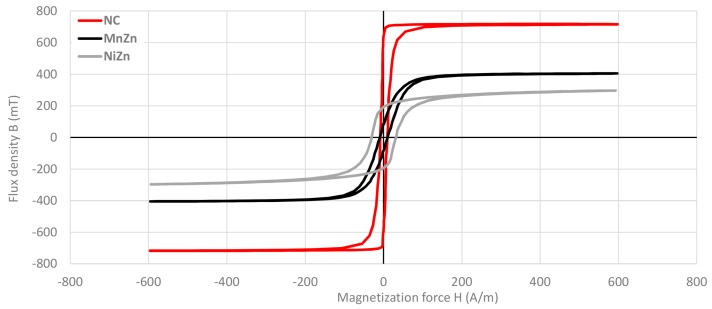
Hysteresis curve measured for NC, MnZn and NiZn cable ferrites.

A great deal of information can be learned about the magnetic properties of a material by studying its hysteresis loop. A hysteresis or B-H loop shows the relationship between the induced magnetic flux density (B) and the magnetizing force (H). The loop is generated by measuring the magnetic flux of a ferromagnetic material while the magnetizing force is changed. As is shown in Figure 5 the hysteresis loop of three cable ferrites is measured with the equipment BsT-Pro B-H-Analyzer. From this graph, it is possible to observe that NC provides the higher magnetic saturation (B_S_), that is in the point where the flux density tends to level off. B_S_ is defined as the maximum flux density attainable in a material at a given temperature and above this value B_S_, it is not possible to further increase B by further increasing H. At B_S_, almost all of the magnetic domains are aligned and an additional increase in the magnetizing force will produce very little increase in magnetic flux. When is reduced to zero, it does not return to zero as it can be seen that some magnetic flux remains in the materials even though the magnetizing force is zero. This is referred to as remnant flux density (B_R_), the point on the graph that indicates the remanence or level of residual magnetism in the materials when H = 0. NC cable ferrite shows the higher value of B_R_. At this point, a part of the magnetic domain remains aligned, whereas the other part has lost its alignment. As the magnetizing force is increased from zero in the opposite direction, the flux density decreases to zero. This is called the point of coercivity of the material (H_C_) and represents the magnetizing force required to remove the residual magnetism from the material. At this point, the reversed magnetizing force has flipped enough of the domains so that the net flux within the material is zero. The maximum value of coercivity is provided by the NiZn cable ferrite so that this composition material needs to be subjected to a larger magnetizing force to reduce its flux density to zero.

Most important parameters of the three cable ferrites are summarized in the Table 2 in order to numerically compare the values observed in the above graphs.

**Figure 6:**
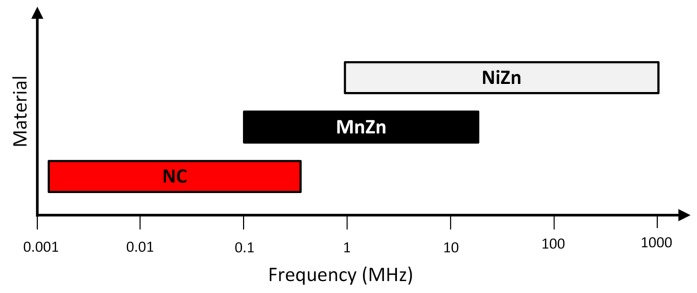
Material composition versus frequency range covered.

Although the permeability is one of the most important parameters which defines the performance of a cable ferrite, other means of characterizing this component are through specifying the magnitude of the impedance versus frequency. The magnitude of the impedance is given by:

(3) $$\left|Z_{F}\right|=\sqrt{R^{2}+{\left(X_{L}\right)}^{2}}$$

where R is the equivalent resistance of the cable ferrite and X_L_ is the impedance of the inductive part. The datasheets of this kind of component generally provide the trace of the ferrite impedance in the frequency range where it is effective or only specify the impedance at several frequency points. The recommended frequency range for various ferrite materials when used in noise suppression applications is shown in Figure 6. As it can be observed, traditional ferrite components such as MnZn and NiZn are available for use over the frequency range of 150 kHz to 1 GHz, whereas NC is intended for covering the low frequency range.

The most common ferrite geometry used in noise-suppression applications is the cylindrical core. A cable ferrite placed around a cable can be effective in reducing both conducted and radiated electromagnetic emission. Thereby, the greater the length of the cylinder, the higher the impedance, so an increase of the core length is equivalent to using several ferrites together. The attenuation or insertion loss provided by a certain cable ferrite depends on the impedance of the system in which this is placed; thus, the cable ferrite should have an impedance higher than the system impedance at the frequency of interest. Hence, they are used most effectively in low-impedance circuits [10].

An interesting characteristic of cable ferrites is the possibility of turning the cable to be filtered around them multiple times. With this technique, the ferrite impedance can be increased proportional to the number of turns squared. However, there is a balance between number of rolls and interwinding capacitance because of the higher the number of turns, the worse the performance at high frequencies. Therefore, the behavior of a cable ferrite can be improved in the 2–150 kHz frequency range, although it should not be overlooked. It is not common to use more than two or three turns. In this research, the characterization is performed by winding one and two turns in the cable ferrites.

### 2.2. Theoretical Insertion Loss Calculation Method

**Figure 7:**
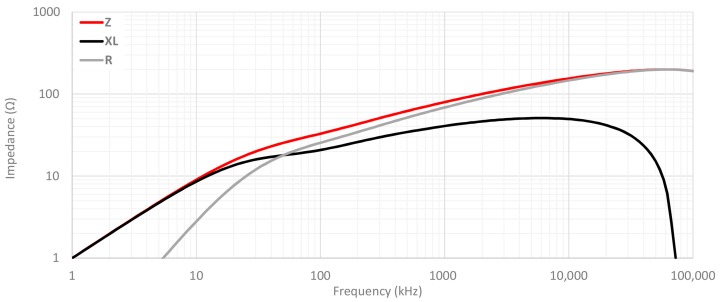
Magnitude of the impedance measured of NC cable ferrite and its components R and X_L_.

One of the most common methods used to characterize a cable ferrite is based on measuring its impedance. The impedance of a cable ferrite can be separated into two components. When this EMC component is used to suppress electromagnetic noise, the loss resistance (R), which represents the losses, needs to be taken into account. Figure 7 illustrates the contribution of loss resistance as well as inductance (X_L_) to the magnitude of the impedance of the NC cable ferrite analyzed in this research. The parameters plotted on this graph have been measured through carrying out the inductance measurement method, but in this case, only one turn is rolled in the component [31]. Thereby, it can be observed in Figure 7 how the inductance is stable in a certain frequency range and show strong frequency range dependence above around 10 kHz. Above 30 MHz the inductance falls sharply, down to zero at around 80 MHz. The loss component (R) grows continuously with frequency and reaches the same value as the X_L_ component at the ferromagnetic resonance frequency. The resistance value rises until MHz range and dominates over the magnitude impedance (Z_F_). Therefore, in this cable ferrite, the absorption part is dominant and the ability to reduce the electromagnetic noise is greater because of the ferromagnetic resonance frequency. This behavior can also be observed in Figure 3 where complex permeability of three materials is represented.

The attenuation that this kind of component is able to provide in a certain conductor is not usually specified in datasheets in contrast to other EMC products such as common-mode-chokes. This is because the insertion attenuation or insertion loss that a cable ferrite is able to provide mainly depends on the impedance of the system in which it is placed. Subsequently, the source impedance (Z_A_) and the load impedance (Z_B_) of the system with electromagnetic interference problems as well as the impedance which the ferrite core introduces in that system (Z_F_) are related in order to obtain an approach of the insertion loss parameter [11]. According to this, taking into account the system impedance and the ferrite impedance in a specific frequency value, the insertion loss in terms of decibels can be theoretically calculated by using this equation:

(4) $$A\left(\mathrm{dB}\right)=20\log\frac{Z_{A}+Z_{F}+Z_{B}}{Z_{A}+Z_{B}}$$

**Figure 8:**
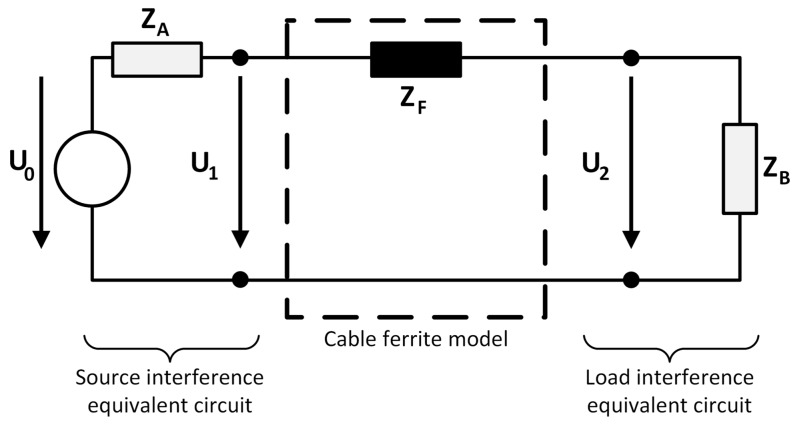
Diagram of source and load equivalents circuits used to determine the insertion loss parameter of a cable ferrite when it is introduced into a system.

The block diagram used to obtain this equation and to analyze the effect of placing a cable ferrite into a certain system is shown in Figure 8.

### 2.3. Insertion Loss Experimental Measurement Setup

**Figure 9:**
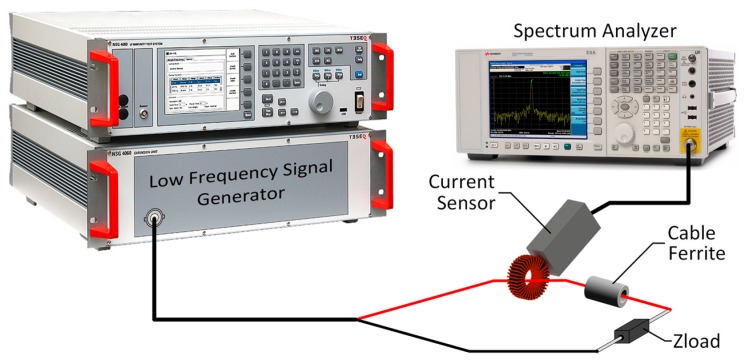
Measurement setup diagram to characterize cable ferrites in the 2–150 kHz frequency range.

The setup used to determine the insertion loss of several cable ferrites is focused on simulating the block diagram shown in Figure 8, where the source and load impedance are known and the parameter to study is the impedance of the ferrite core. Thereby, the setup designed to simulate that diagram is shown in Figure 9 and it consists of:

- A low frequency immunity test system based on the NSG-4060/1 Low Frequency Signal Generator (TESEQ, Luterbach, Switzerland) with 50 Ω of output impedance is employed to generate the reference signal that crosses the cable ferrite. Thus, this 50 Ω output resistance represents the Z_A_ in the insertions loss block diagram. This sine wave generator and integrated power amplifier consists of a signal generator able to provide signals for the frequency range of 15 Hz to 150 kHz. The probe connected to this generator separates the signal and the ground terminals to place the cable ferrite only in the signal path. The main objective of this part of the setup is focused on characterizing the performance of cable ferrites in this range of frequencies through simulating the electromagnetic noise which can appear in a real system.
- The N9010A Spectrum Analyzer (Keysight, Santa Rosa, CA, USA) is used both for measuring the amplitude of the signal generated as a reference and the signal when the cable ferrite is placed around the cable. This equipment makes it possible to analyze the attenuation provided by each kind of cable ferrite in the range of 2–150 kHz. The measurement is carried out with a low frequency current probe which measures the signal before and after placing the cable ferrite.
- Different resistance loads are employed to evaluate the performance and robustness of ferrites. In order to analyze the characteristics of cable ferrites with different values of load impedance, a PCB (Printed Circuit Board) which holds several values of impedance has been designed. This circuit is intended for switching among four different values of Z_B_ 5 Ω, 50 Ω, 100 Ω and 1000 Ω. Since the performance of cable ferrites relies on the load impedance, this part of the setup allows us to study the behavior of each material composition in systems with different load values.

Therefore, the test is carried out by attaching the current probe in the signal terminal of the signal generator probe and measuring the noise emitted with the Spectrum Analyzer. This is tuned to measure the interference signal generated by the Low Frequency Signal Generator in the 2–150 kHz band. This measurement process is repeated twice: only measuring the signal terminal, and measuring with the cable ferrite placed around the signal terminal.

Subsequently, both stored signals will be compared and the attenuation rate or insertion loss parameter of a certain cable ferrite can be determined by subtracting the reference signal with the signal measured after attaching the EMC component. Both measurements are acquired with the max-hold option enabled in the Spectrum Analyzer in order to compare them.

## 3. Results and Discussion

The results presented in this part, correspond to the analysis of the acquired data through the theoretical and experimental methods described above. Firstly, the insertion loss parameter of the three cable ferrites under test has been calculated accordingly with the described theoretical method. Subsequently, the insertion loss parameter measured by following the experimental setup explained are plotted in order to compare both methods and characterize the three cable ferrites at low frequency range. Considering this, it is possible to evaluate the performance of nanocrystalline cable ferrite to provide a solution to filter the electromagnetic noise in the 2–150 kHz spectrum range and compare it with another material analyzed.

### 3.1. Theoretical Insertion Loss Results

**Figure 10:**
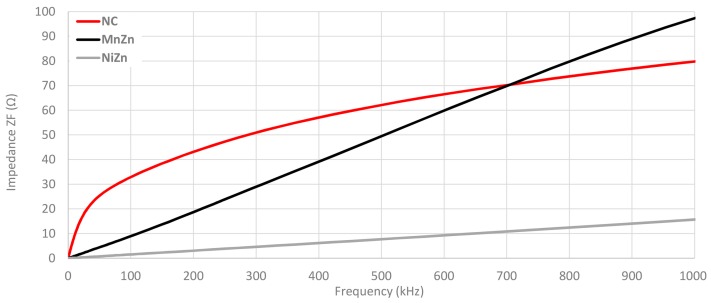
Impedance comparison of the three cable ferrites measured with 1 turn.

As it has been described in Section 2.2, theoretical insertion loss parameter is calculated from the magnitude of the impedance. Figure 10 shows that the impedance of NC cable ferrite is higher than MnZn and NiZn components until 700 kHz. From this frequency, MnZn cable ferrite provides a greater performance, although its initial magnitude impedance is lower, the angle of slope of its trace is higher than NC. With regard to NiZn cable ferrite trace, it can be observed how its impedance is much lower if this is compared with NC and MnZn materials because it is destined to a higher frequency range. 

**Figure 11:**
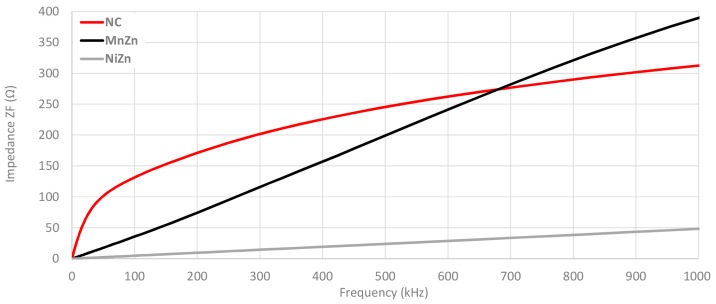
Impedance comparison of the three cable ferrites measured with 2 turns.

This measurement has been repeated, but in this case, by using two turns as shown in Figure 11. This change generates higher values of magnitude impedance in the three traces and a shift in the cross point of NC and MnZn traces. Thereby, if the 100 kHz point is taken as a reference, three traces have been increased about four times (the number of turns squared). Specifically, in the case of NC from 33.2 Ω to 132.7 Ω, MnZn 9.2 Ω to 36.6 Ω and NiZn cable ferrite from 1.6 Ω to 5.0 Ω. As regards the cross point between NC and MnZn traces, it has been moved from 705 kHz to 680 kHz when two turns are wound in the cable ferrites.

**Figure 12:**
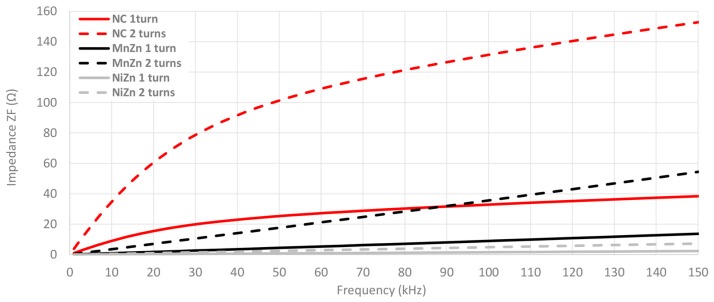
Impedance comparison of the three cable ferrites measured with 1 and 2 turns in the low frequency range.

A zoom in on 2–150 kHz frequency range is performed as shown in Figure 12 in order to analyze in detail the impedance provided for each cable ferrite when one and two turns are wound. This graph demonstrates the huge difference among the impedance provided by NC cable ferrite and the others. If 100 kHz is taken as a reference, NC provides 24.0 Ω more than MnZn and 31.6 Ω than NiZn cable ferrites with one turn. Considering two turns, NC impedance is 96.1 Ω higher than MnZn and 127.7 Ω. It can be also observed that NC with one turn is even higher than MnZn with two turns until 88 kHz.

**Figure 13:**
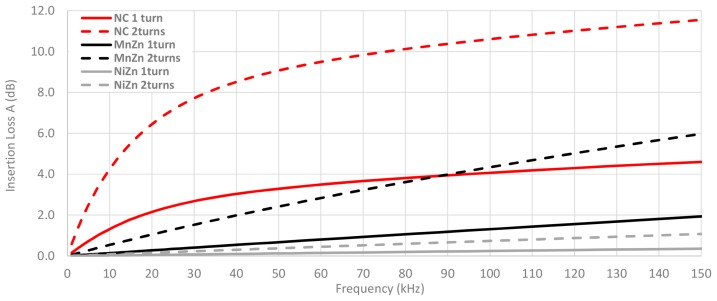
Theoretical insertion loss comparison depending on the material composition calculated with a 5 Ω load and winding 1 and 2 turns.

Once the magnitude impedance has been analyzed, the insertion loss parameter is calculated through Equation (4) from Z_F_ measured of each cable ferrite, Z_A_ = 50 Ω and Z_B_ = 5 Ω. Figure 13 shows the calculated or theoretical insertion loss parameter of the three cable ferrites with one and two turns of cable are wound. As illustrated in Figure 13, NC is again the composition that provides the best performance to attenuate the electromagnetic noise until 150 kHz. Moreover, all insertion loss traces are proportional to the magnitude impedance represented above. Taking 100 kHz as a reference point, NC attenuates 2.8 dB more than MnZn and 3.8 dB than NiZn cable ferrites with one turn. In the case of two turns, NC attenuation is 6.3 dB higher than MnZn and 9.9 dB than NiZn cable ferrites.

**Figure 14:**
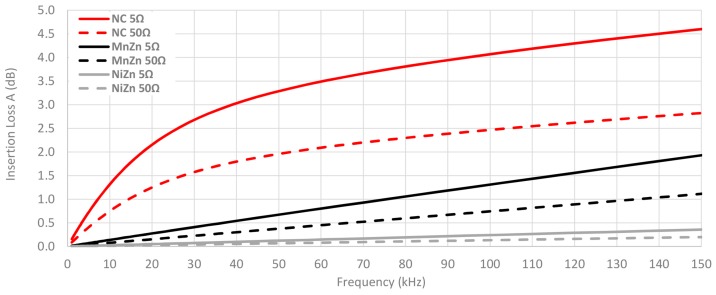
Theoretical insertion loss comparison depending on the material composition calculated with 5 Ω and 50 Ω loads by winding 1 turn.

Another factor to consider is the performance of cable ferrites in systems with different impedance and, thus, it could be interesting to study the insertion loss parameter by including several values of Z_A_ and/or Z_B_ in Equation (4). Consequently, Figure 14 shows a comparison among the insertion loss parameter calculated from Z_F_ measured of each cable ferrite with one turn, setting Z_A_ = 50 Ω and Z_B_ = 5 Ω or Z_B_ = 50 Ω. This chart illustrates how, the higher the system output impedance in which the cable ferrite is included, the lower the insertion loss that is able to provide the cable ferrite. At the 100 kHz frequency point, there is a difference of 1.6 dB between the NC traces with 5 Ω and 50 Ω, 0.5 dB in the case of MnZn traces and 0.2 dB for NiZn traces.

**Figure 15:**
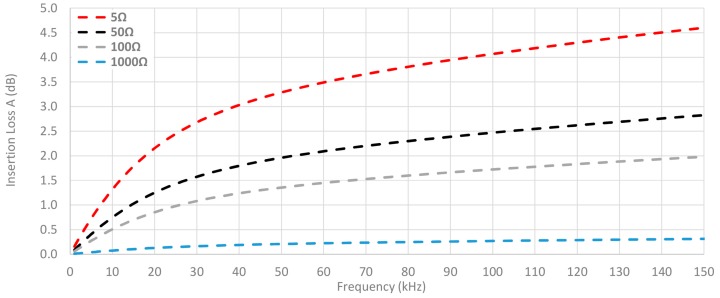
Theoretical insertion loss comparison depending on load calculated with 5 Ω, 50 Ω, 100 Ω and 1000 Ω loads by winding 1 turn in the NC cable ferrite.

This connection between the system impedance and the ability of cable ferrites to attenuate the interferences is displayed in Figure 15, where the insertion loss is calculated by setting Z_A_ = 50 Ω and Z_B_ is switched among the next values: 5 Ω, 50 Ω, 100 Ω and 1000 Ω. The material selected to analyze this connection is the NC because it provides the best performance in this frequency range. Thereby, this graph shows that the theoretical insertion loss is much reduced when the impedance of the system is close to 1 kΩ. However, if the system has an input impedance of 50 Ω and an output impedance less than 100 Ω the NC cable ferrite with one turn can provide useful behavior.

### 3.2. Experimental Insertion Loss Results

**Figure 16:**
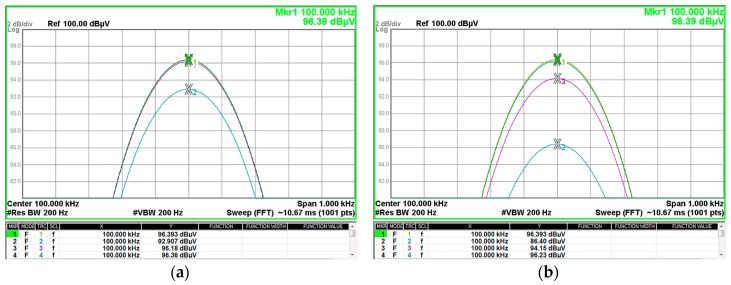
Experimental attenuation measured with a spectrum analyzer for each cable ferrite material with a 5 Ω (Reference: trace 1 yellow, NC: trace 2 blue, MnZn: trace 3 purple, NiZn: trace 4 green): (**a**) Signal measured after placing each cable ferrite with one turn; (**b**) Signal measured after placing each cable ferrite with two turns.

The experimental insertion loss is measured by following the procedure explained in Section 2.3. Figure 16 shows an example of the measurement method used to determine the insertion loss parameter with one turn (a) and two turns (b). Experimental insertion loss is determined by subtracting to the trace 1 (yellow), which represents the reference before placing any cable ferrite in the system, one of the other three traces. Trace 2 (blue) represents the signal measured after placing the NC cable ferrite, trace 3 (purple) corresponds to MnZn and trace 4 (blue) is acquired when the NiZn cable ferrite is placed. Therefore, the insertion loss of NC cable ferrite at 100 kHz is given by subtracting markers 1 and 2 represented in the table below the spectrum in (a) in the case of one turn and (b) in the case of two turns. As a result of this step, the insertion loss obtained for the NC cable ferrite is 3.486 dB with one turn and 9.993 dB when the cable ferrite is wound with two turns. Consequently, by extrapolating this method to the whole frequency range of 2–150 kHz it is possible to obtain the performance of each material in terms of attenuation ratio.

**Figure 17:**
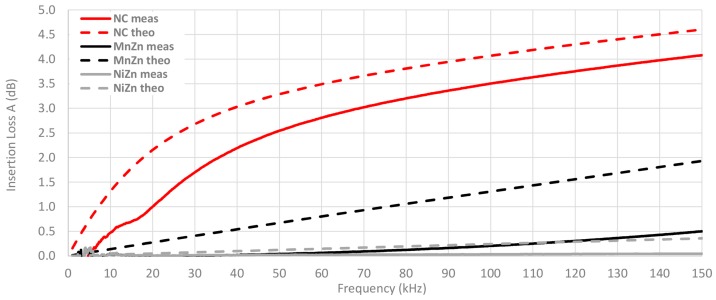
Experimental insertion loss comparison depending on the material composition calculated with a 5 Ω load by winding 1 turn.

A comparison between the theoretical insertion loss and the insertion loss measured with the three material cable ferrites with one turn wound is shown in Figure 17. The measured value of NC cable ferrite is 1.2 dB lower than the theoretical NC trace at 20 kHz, although, at 100 kHz this difference is reduced up to 0.6 dB. Nevertheless, in the case of MnZn and NiZn, the higher the frequency, the higher the difference between theoretical and experimental insertion loss. Hence, the NC traces trend is very similar but the theoretical and experimental traces of MnZn and NiZn do not follow the same trend.

**Figure 18:**
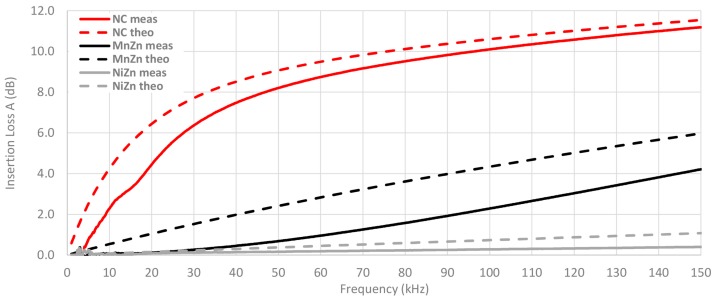
Experimental insertion loss comparison depending on the material composition calculated with a 5 Ω load by winding 1 turn.

Figure 18 shows the same comparison as the last graph but in this case, the number of turns wound with the cable in the cable ferrite is two. It can be observed how the difference between theoretical and measured traces is lower with two turns. At 20 kHz, the NC insertion loss measured is 1.8 dB lower than theoretical value and this difference is reduced up to in 0.6 dB at 100 kHz. Thus, the relative error has been reduced taking into account that the attenuation ratio has been almost tripled (from 3.5 dB to 10.1 dB at 100 kHz in the case of measured insertion loss traces). With regard to MnZn, the trend of measured insertion loss trace has been modified and the difference with the theoretical trace has been reduced. This effect can also be observed in the NiZn traces, although to a lesser extent than NC and MnZn.

**Figure 19:**
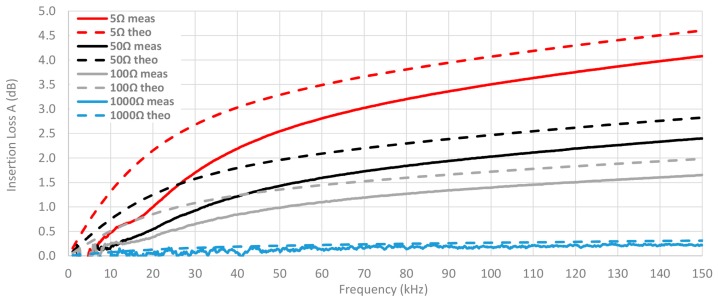
Comparison between experimental and theoretical insertion loss of NC cable ferrite depending on the load by winding 1 turn.

Another point to take into account is the connection between the impedance of the system where the cable ferrite is placed and the insertion loss that it is able to provide. In order to evaluate this, the insertion loss of the NC cable ferrite is measured by setting Z_A_ = 50 Ω and modifying Z_B_ among the next values: 5 Ω, 50 Ω, 100 Ω and 1000 Ω. Figure 19 shows this comparison combined with the theoretical insertion loss traces showed in the Figure 15. According to the graph, traces that represent measured insertion loss are lower than theoretical traces, although the trend of both is very similar. Just as for the theoretical NC trace, the experimental insertion loss measured shown in this graph is insignificant when the system impedance is close to 1000 Ω.

**Figure 20:**
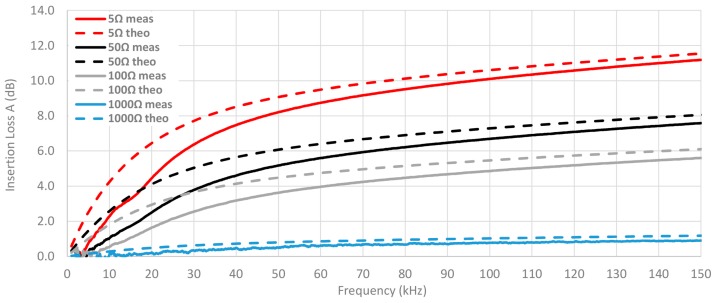
Comparison between experimental and theoretical insertion loss of NC cable ferrite depending on the load by winding 2 turns.

Figure 20 shows the same comparison as Figure 19, but in this case, two turns are wound in the NC cable ferrite. Therefore, the difference between measured and theoretical insertion loss is reduced regardless of the system impedance. Moreover, it can be observed that the higher the frequency, the lower the difference between theoretical and experimental insertion loss.

**Figure 21:**
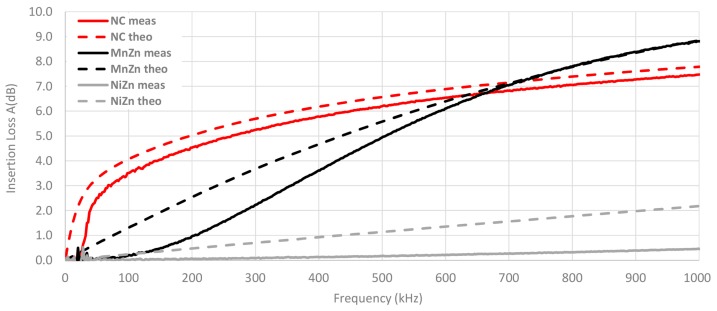
Comparison between experimental and theoretical insertion loss of different cable ferrites depending on the material composition by winding 1 turn at higher frequencies.

Considering that the difference between theoretical and experimental insertion loss traces is lower at high frequencies, the insertion loss has been measured with a general purpose signal generator with the aim of obtaining a qualitative trend up to 1 MHz. This measurement has been carried out for the three cable ferrites with one turn and compared to the calculated insertion loss in Figure 21. Therefore, it is possible to observe that the difference between NC traces is lower at high frequency and MnZn traces match at 700 kHz. Nevertheless, the difference between NiZn traces is higher at 1 MHz.

## 4. Conclusions

A characterization of a novel nanocrystalline cable ferrite and a comparison with traditional materials has been performed from the point of view of magnetic properties, manufacturing process and EMI suppression ability.

The results presented in this contribution demonstrate the suitability of cable ferrites based on nanocrystalline composition in the 2–150 kHz frequency range in comparison with others based on MnZn or NiZn. This has been verified theoretically through calculating the insertion loss from the measured impedance of each cable ferrite material and taking into account the system impedance where the component is placed. A way of determining the attenuation ratio or insertion loss through an experimental setup has been proposed and these measurements have been matched with the theoretical insertion loss in order to check the accuracy of this technique. Thereby, the measurements performed both theoretical and experimental, indicate that NC provides greater losses at low frequencies than MnZn and NiZn as shown the relative permeability graph.

The system impedance parameter has been modified to study the performance of cable ferrites with several output impedances and the results show that the attenuation ratio is too low in systems with an impedance close to 1000 Ω. Likewise, the analysis has been done with one and two turns around the cable ferrites in order to take into account the performance of each material when the number of turns is increased. These last measurements show that the difference between measured and calculated insertion loss is reduced when the winding is increased. This could take place because the inductance and resistance parts of the magnitude impedance are shifted to lower frequencies. Thus, it can generate a change in the trend of the insertion loss measured trace. This difference between theoretical and experimental data can be also analyzed from the point of view of permeability because, as shown Figure 21, theoretical and experimental values are nearer from the frequency resonance point in which losses are higher. In the case of NC, this takes place at 45 kHz and from this point, it is possible to observe a reduction in the difference between theoretical and experimental traces. With regard to MnZn, there is a nearness between theoretical and experimental traces as the frequency increases and, both traces match from the frequency resonance point up to higher frequencies. The same effect could be observed at 10 MHz where the absorption part starts being more dominant in the case of NiZn cable ferrite. Consequently, a new line of research can be carried out from the results of this contribution, because this setup can be adapted to determine the insertion loss with the aim of evaluating cable ferrites at higher frequencies. This method could make it possible to study the accuracy of the insertion loss mathematical expression in order to know if R (µ′′) and X_L_ (µ′) should be considered with a different weight in this theoretical approximation.

## Figures and Tables

**Table 1:** Lists of cable ferrites used in this contribution.

Ferrite Part Number	Magnetic Material	External Diameter (mm)	Internal Diameter (mm)	Height (mm)
M-4304-02	NC	18.9	12.9	27.7
74277255	MnZn	18.6	10.2	28.5
74270055	NiZn	18.6	10.2	28.5

**Table 2:** Magnetic properties of cable ferrites.

Ferrite PN	Material	Initial Perm ^1^	Curie Temp ^2^	Sat. Flux Density ^3^	Sat. Field Density ^4^	Coercivity ^5^	Resonance Frequency
M-4304-02	NC	89,400	150 °C	717.8 mT	222.4 A/m	8.5 A/m	33 kHz
74277255	MnZn	5638	>180 °C	404.8 mT	477.4 A/m	9.2 A/m	875 kHz
74270055	NiZn	783	135 °C	296.7 mT	567.9 A/m	30.9 A/m	16.5 MHz

Test conditions: ^1^ 10 kHz, <0.5 mT, 25 °C; ^2^ 10 kHz, <0.5 mT; ^3,4,5^ 10 kHz, 600 A/m, 25 °C.
